# Spatio‐temporal investigation of reported cases of animal rabies in Ghana from 2010 to 2017

**DOI:** 10.1002/vms3.1282

**Published:** 2023-09-23

**Authors:** Paa Kobina Turkson

**Affiliations:** ^1^ School of Veterinary Medicine University of Ghana Accra Ghana

**Keywords:** dogs, Ghana, rabies, spatio‐temporal analyses

## Abstract

**Background:**

Rabies is a zoonotic disease transmitted mainly by animals, especially dogs.

**Objective:**

The aim of the article was to examine reported cases of animal rabies in Ghana for trends to provide information that could be helpful to control the disease.

**Method:**

Retrospective analyses of reported cases of rabies in Ghana from 2010 to 2017.

**Results:**

In all, 328 rabies cases were recorded in animals in the period under review. The predominant species involved were dogs (299; 91.2%) and cats (12; 3.7%). Other species included pigs (4; 1.2%), goats (4; 1.2%), monkeys (4; 1.2%), sheep (2; 0.6%), bats (2; 0.6%) and cattle (1; 0.3%). The numbers of reported cases in animals were markedly higher than those in humans except in 2013 and 2017. There was a positive but weak correlation between cases in animals and humans which could be due to lack of reporting collaboration between institutions responsible for these. Greater Accra and Ashanti Regions were identified as hotspots in the period under review, while January and August were the months with the highest peaks for cases reported. Cases reported in rainy season were significantly higher than those in dry season. Poisson regression for spatio‐temporal analyses showed no statistical significance in predicting number of rabies cases (response variable) from year, month, season, region and affected species (predictor variables).

**Conclusion:**

Rabies remained endemic in Ghana during 2010–2017 with cases reported in nearly every month of the year during this period. There was a significant seasonal pattern with higher proportion of cases reported in the rainy/wet season compared to the dry season.

## Graphical abstract

Rabies is endemic in Ghana. Dogs were the species most affected. There was a significant difference in number of reported cases of rabies in the rainy season (April–October) compared to cases in the dry season (November–March).

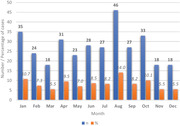

## 1 INTRODUCTION

Rabies is a fatal zoonotic disease in humans transmitted by animals, especially bites from pets. It is transmitted mainly by dogs and is caused by rabies virus in the family Rhabdoviridae and genus *Lyssavirus*. Rabies is endemic/enzootic in Ghana. Several studies have reported on rabies in Ghana. These include epidemiology and control measures (Addy, 1985; Alonge & Abu, 1984; Belcher et al., 1976; Lopes et al., 2018); surveillance systems (Afakye et al., 2016; Guri et al., 2020); human rabies outbreaks (Amoako et al., 2021; Apanga et al., 2016; Dsane‐Aidoo et al., 2022; Laryea et al., 2017; Punguyire et al., 2017); dog bites and rabies (Abuh et al., 2017; Addai & Nuertey, 2018; Adomako et al., 2018; Dsane‐Aidoo et al., 2022; Eliezer, 2016; Kenu et al., 2018; Korash & Ameme, 2017; Punguyire et al., 2017, 2020); dog population structure (Tasiame et al., 2019); dog‐associated pig rabies (Tasiame et al., 2016); rabies virus in dogs slaughtered for meat consumption (Tasiame et al., 2021); knowledge, attitudes, perceptions, practices and beliefs (Awuni et al., 2019; Tettey, 1998; Turkson & Wi‐Afedzi, 2020; Vetsi et al., 2021) and lineage of rabies virus in Ghana (Hayman et al., 2011). However, no studies have reported on the space–time pattern of the disease countrywide on a national level.

Rabies is a public health concern in Ghana due to a large stray dog population, weak surveillance system and limited laboratory and vaccines supply in the country (Afakye et al., 2016; Awuni et al., 2019; Kenu et al., 2018; Laryea et al., 2017; Ministry of Food and Agriculture, Veterinary Services Directorate [MOFA‐VSD], 2021; Punguyire et al., 2017; Tettey, 1998). There have been challenges to rabies control in Ghana. Animal rabies outbreaks are usually associated with outbreaks in humans (Nang‐Lazuma, 2015). The available data on rabies are inadequate due to poor disease‐reporting systems (Turkson & Wi‐Afedzi, 2020). The lack of reliable data and systematic analysis of available data continue to make rabies a neglected disease in Ghana (Turkson & Wi‐Afedzi, 2020). Monitoring of human rabies outbreaks in Ghana is inadequate due to poor coordination between institutions responsible for human and animal health (Turkson & Wi‐Afedzi, 2020), resulting in lack of coherence or congruence in data on rabies reported from these sources.

Published information in literature on the distribution of rabies cases in Ghana is non‐existent or, at best, lacking. This article presents the spatial and temporal distribution of reported cases of rabies in Ghana in animals over an 8‐year period (2010–2017), allowing outbreaks to be distinguished from each other, helping to understand the distribution of the reported cases and identifying hotspots or trends or patterns that could be useful to control rabies in animals in Ghana.

## 2 MATERIALS AND METHODS

This was a retrospective study on reported rabies cases in Ghana over an 8‐year period (2010–2017) obtained from the Veterinary Services Directorate of the Ministry of Food and Agriculture in Ghana. It was the only time period with available useful data comprising of dates of reported suspected cases, number of suspected cases on monthly basis from 2010 to 2017, species of animals affected, numbers susceptible, numbers of animals infected, georeferenced locations (latitudes and longitudes) of cases in animals using handheld GPS equipment and reported number of cases of rabies in humans. For the country as a whole the wet season was from April to October, whereas the dry season was from November to March. Data on human rabies cases for 2010–2017 were sourced from Rabies in West Africa (RIWA), Ghana office.

A passive surveillance system is used by the Veterinary Services Directorate for animal rabies reporting in Ghana. Suspected cases are reported to Accra and samples sent to Veterinary Laboratories in Accra and Tamale. The reporting system was observed to be poor and could lead to underreporting.

## 3 DATA ANALYSIS

Data analyses were done using Microsoft Excel. Georeferenced locations with reported rabies cases were used to plot maps in ArcMap version 10.8.1 (ESRI). In few cases where coordinates for locations were missing, the coordinates of the nearest town obtained from Google Maps were used. The Poisson regression analysis in SPSS version 24 was used to model spatial and temporal correlation between observations to predict future cases. The dependent variable was number of cases, whereas the predictor variables were year, month, season, region and species of animals affected. The proportion of cases in the rainy months was compared with that in the dry months in a one‐sample *Z* test using MedCalc calculator (MedCalc Software Ltd, 2023) available online.

No ethical clearance was obtained because the data did not have any identifiers or information traceable to specific individuals or groups of humans or animals.

## 4 LIMITATIONS

The data available were for reported suspected cases and did not have information on clinically confirmed or laboratory‐confirmed cases. There could, therefore, have been reporting bias and lack of confirmation of the cases.

The data were limited to 2010–2017 because these were the years with useful data available. For the years before 2010 or after 2017, no or incomplete data were available for use. Some areas or regions and some months had zero reports raising issues as to whether there were actually no cases for those areas or months or no reports were received. The exact reasons could not be ascertained.

## 5 RESULTS

**FIGURE 1:**
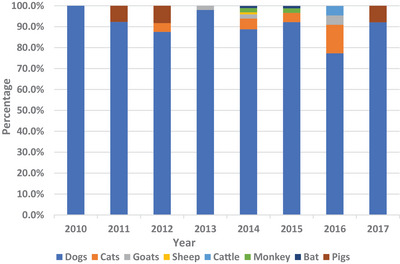
Proportions of reported cases of rabies in Ghana from 2010 to 2017 based on animal species.

The total number of cases of rabies reported in the period was 328. The predominant species involved were dogs (299; 91.2%) and cats (12; 3.7%). Other species included goats (4; 1.2%), monkeys (4; 1.2%), pigs (4; 1.2%), sheep (2; 0.6%), bats (2; 0.6%) and cattle (1; 0.3%). Figure 1 provides the proportions of reported rabies cases according to species from 2010 to 2017.

**FIGURE 2:**
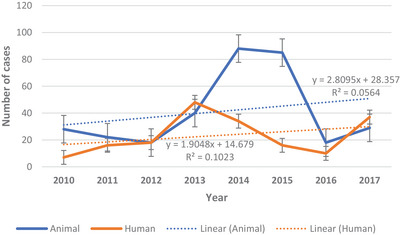
Annual cases of rabies in animals and humans in Ghana from 2010 to 2017.

Distributions of reported rabies cases on yearly basis in animals and humans are presented as scatterplot in Figure 2. Generally, a number of reported cases in animals were markedly higher than those in humans apart from 2012 (where they were similar) and 2013 and 2017 (where they were lower than those in humans). The correlation between number of cases in humans and animals over the period was positive but weak (Pearson correlation coefficient of 0.26).

**FIGURE 3:**
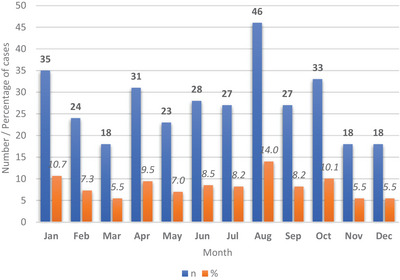
Total monthly number and proportion of cases of rabies in animals in Ghana from 2010 to 2017.

Figure 3 presents the composite monthly proportion of cases of rabies in animals in Ghana from 2010 to 2017, showing the highest in August (14.0%) followed by January (10.7%). The lowest proportion (5.5%) was in March, November and December. The mean monthly number of cases followed a similar pattern with a minimum of 2.3 in March, November and December and a maximum of 5.8 in August over the period under review. The mean numbers for other months were January 4.4; February 3.0; April 3.9; May 2.9; June 3.5; July 3.4; September 3.4 and October 4.1.

Attached are supplementary files Figures [Link], [Link], [Link], [Link], [Link], [Link], [Link], [Link] which show the distribution of annual cases of rabies from 2010 to 2017.

For seasonal prevalence, a higher proportion of cases (64.9%) was seen in the rainy season (April–October) compared to that in the dry season (November–March) (35.1%). The difference in proportions was significant (*Z*‐statistic 5.63; *p* < 0.0001; 95% confidence interval: 60.1%–70.7%).

**FIGURE 4:**
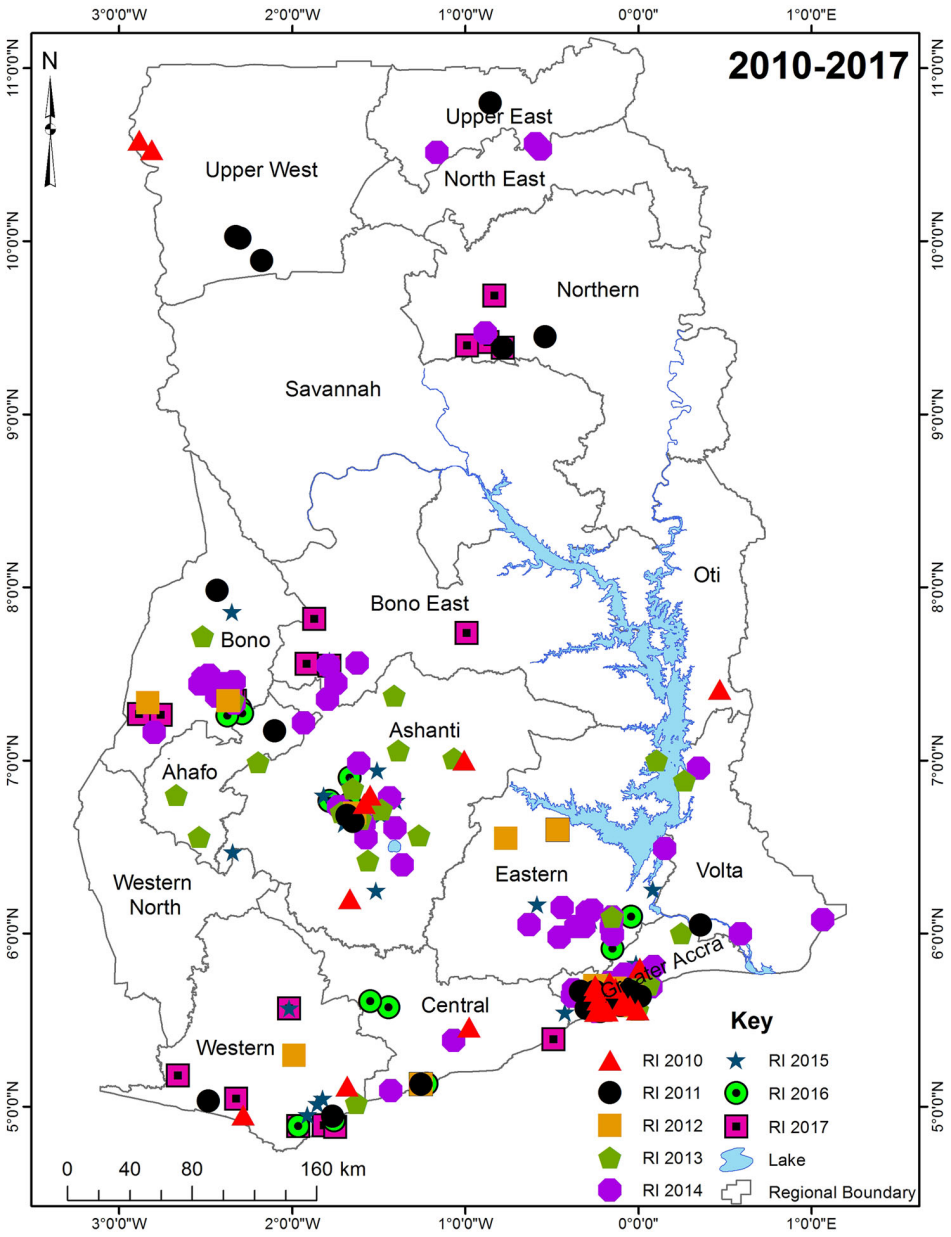
Spatial distribution of rabies cases in Ghana from 2010 to 2017. RI, rabies incidence.

Figure 4 presents spatial distribution of cases from 2010 to 2017. No clear patterns were evident apart from Greater Accra and Ashanti (except in 2017) Regions consistently reporting higher number of cases over the period.

Attached are supplementary files Figures [Link], [Link], [Link], [Link], [Link], [Link], [Link], [Link] showing the spatial distribution of cases of rabies from 2010 to 2017.

A Poisson regression modelling, used as a spatio‐temporal analytical tool, showed that none of the predictor variables (year, month, season, species affected and region of cases), had any statistically significant effect on the response variable (number of cases reported).

## 6 DISCUSSION

The finding of dogs as the species having most cases of rabies in Ghana supports a World Health Organisation report that the major vector of rabies in Ghana is the dog (World Health Organisation [WHO], 1992). Further, the WHO reported that between 1981 and 1991 other species affected in Ghana were cattle and sheep. From 1970 to 1982, dogs were responsible for 98.7% of rabies cases in Ghana, cats 0.07% and cattle 0.7% (Addy, 1985). Dog bites accounted for 96.5% (*n* = 994), cats 1.8% (*n* = 18), non‐human primates 1.2% (*n* = 12) and human bites 0.6% (*n* = 6) in a study of human rabies cases in Accra between 2013 and 2016 (Addai & Nuertey, 2020). In our study, dogs were involved in 91.2% of reported cases, with cats contributing 3.7%. In Ghana, rabies virus has been identified in dogs, cats, monkeys, pigs (Tasiame et al., 2019), cattle, sheep and goats (Veterinary Services Directorate [VSD], 2018). Similar species were identified in our study in addition to bats. However, the report of bats is questionable since there are no known rabies virus variants in bats in Africa. Domestic dogs were responsible for 94% of human rabies through bites in Ghana (Tasiame et al., 2019). Therefore, it is necessary to intensify rabies prevention and control measures in dogs, if progress is to be made.

With regard to spatial distribution of cases, no particular pattern or trend was evident. Region of cases was not significant in the Poisson regression done. However, cases seemed to have been more commonly reported in the southern half of the country compared to the northern half for most years. This could be due to willingness to report dog bite cases.

From 1999 to 2012, 685 suspected dog rabies outbreaks were reported in the country (VSD, 2018). Rabies is under‐reported in many countries worldwide (Fooks, 2005), in Africa (due to the absence of reliable surveillance data; Adesina et al., 2020) and in Ghana (Turkson & Wi‐Afedzi, 2020). There is very little systematically collected and analysed data on dog bites and rabies, a major reason why the disease is neglected (Afakye et al., 2016; Apanga et al., 2016). The lack of reliable data and systematic analysis of available data continues to make rabies a neglected disease in Ghanaian society (Abuh et al., 2017). Underreporting, poor surveillance and a weak One Health approach to surveillance are said to be responsible for the often incompleteness of reports on rabies (Guri et al., 2020), as was also evident in the present study. Major discrepancies indicating poor surveillance, reporting and cooperation among national, international and global authorities have been identified in analyses of reported data (Nel, 2013). Rabies surveillance data in Ghana are poor. According to Minhaj et al. (2023), for a country to be designated as having adequate surveillance, at least one suspected rabies animal should be tested for every 100,000 persons per year. This suggested that Ghana should be testing over 330 suspected rabies cases per year which is not the case. The true extent of rabies occurrence is unknown since officially recorded figures of laboratory‐confirmed cases only provide an indication of what may be happening.

A number of factors have been identified as contributing to poor or inaccurate reporting of rabies cases. These include non‐presentation and/or reporting of suspected cases, non‐availability of local district diagnostic facilities, poor packaging and transportation of specimens to diagnostic laboratories and lack of awareness among the public (VSD, 2018). These need to be addressed holistically, if progress is to be made.

Dog vaccination is widely recommended as the prevention and control approach to rabies outbreaks. The increase in rabies cases in many parts of Ghana has been linked to the unwillingness of pet owners to vaccinate animals and the presence of many dogs whose real owners do not care for them and are, therefore, considered stray (Addo, 2012). In addition, previous control measures involving dog vaccination and removal of stray dogs have not been continuous or sustained leading to failures. The coverage of dog vaccination in Ghana from 2000 to 2013 was between 5% and 30% (RIWA Ghana, 2016) and 5.5% between 2014 and 2018 (Guri et al., 2020). These are far below the 70% required to achieve ‘herd immunity’, a ‘*conditio sine qua non’* for the effective control of the disease.

There seems, generally, to exist a disparity between the number of rabies cases between human health and veterinary services reports on rabies in animals and humans, respectively (Figure 2). The finding of weak positive correlation between outbreaks in humans and animals appears anomalous and may be due to issues of reliability and congruence of data from animal and human health services. There are parallel, separate and uncoordinated rabies surveillance systems in Ghana (Adomako et al., 2018), which are passive in nature and tend to depend on voluntary reports by dog owners or people bitten by dogs. The two independent systems of disease reporting use different sets of information with no common platform for data‐sharing and reconciliation to ensure harmonised disease information collection, management and dissemination (Adomako et al., 2018). In the western region of Ghana, close collaboration between animal and human health services resulted in data on reported dog bites, animal and human rabies cases provided by the two bodies being similar (Turkson & Wi‐Afedzi, 2020), indicating that this is possible.

The efforts in Ghana to eliminate rabies and other zoonotic diseases remain undermined by the lack of reliable data and uncoordinated, community‐based surveillance. This problem extends to the global level, where disparities in the number of cases of rabies reported to the WHO and the World Organization for Animal Health (WOAH) continue to persist (Adomako et al., 2018). Rabies is often handled separately by health and veterinary authorities, and there is regular confusion as to who is responsible for controlling the disease (Nel, 2013). For any breakthrough to be made, these need to be addressed. The adoption and implementation of a One Health approach may provide a solution (Abbas et al., 2011, Adomako et al., 2018; Acharya et al., 2020). A National One Health Policy with the goal of safeguarding human, animal and environmental health in Ghana has been drafted (National Disaster Management Organization, Ministry of Health, Ministry of Food and Agriculture and Ministry of Environment, Science, Technology and Innovation [NADMO‐MOH‐MOFA‐MESTI], 2020). It proposes improving governance and leadership; advancing advocacy and communication; improving research through collaborations on One Health; enhancing capacity‐building to facilitate implementation of the One Health Policy agenda; improving food security and food safety; improving disease prevention, surveillance, response and recovery; ensuring environmental health and addressing transnational border issues to minimise health‐related risks. It has been argued that for One Health to be operationalised, strong intersectoral collaborations are necessary (Arredondo et al., 2021). The agencies involved in rabies disease surveillance and control in humans and animals in Ghana must work together to achieve the needed results.

## 7 CONCLUSION

Rabies remained endemic in Ghana during 2010–2017 with cases reported in nearly every month of the year during this period. There was a significant seasonal pattern with higher proportion of cases reported in the rainy/wet season compared to the dry season. Analyses of reports of dog bites and rabies cases in dogs and other animals could help in directing resources and efforts aimed at preventing and controlling rabies outbreaks in animals and humans in the light of poor or limited funding available in the country.

## AUTHOR CONTRIBUTIONS

*Conceptualisation; data curation; formal analysis; funding acquisition; investigation; methodology; project administration; resources; supervision; validation; visualisation; writing – original draft; writing – review and editing*: Paa Kobina Turkson.

## CONFLICT OF INTEREST STATEMENT

The author declares no conflict of interest regarding the publication of this article.

## FUNDING INFORMATION

No sponsorship was received for the collection of data and writing of the article.

## ETHICS STATEMENT

The authors confirm that the ethical policies of the journal, as noted on the journal's author guidelines page have been adhered to. No ethical approval was required as this is an analysis of disease outbreak reports using aggregated data and no personal identifiers.

### PEER REVIEW

The peer review history for this article is available at https://www.webofscience.com/api/gateway/wos/peer‐review/10.1002/vms3.1282.

## Supporting information

Supporting Information

Supporting Information

Supporting Information

Supporting Information

Supporting Information

Supporting Information

Supporting Information

Supporting Information

Supporting Information

Supporting Information

Supporting Information

Supporting Information

Supporting Information

Supporting Information

Supporting Information

Supporting Information

## Data Availability

The data used are available as records from the Veterinary Services Directorate, Accra. They are not available online.
