# Supplementary material for: Spatio‐temporal investigation of reported cases of animal rabies in Ghana from 2010 to 2017
Source: Vet Med Sci. 2023 Sep 23;9(6):2559–65. doi: 10.1002/vms3.1282 (PMC10650226; doi:10.1002/vms3.1282)
Supplement: Supplementary file 1 — Supporting Information [file VMS3-9-2559-s016.docx]

**SUPPLEMENTARY INFORMATION**

Figures S1 to S8 present the cases of rabies in animals on monthly basis from 2010 to 2017 used in preparing Figure 3. No particular pattern or trend was evident.

Figure S1: Monthly cases of rabies in animals in Ghana in 2010.
