# Supplementary material for: Spatio‐temporal investigation of reported cases of animal rabies in Ghana from 2010 to 2017
Source: Vet Med Sci. 2023 Sep 23;9(6):2559–65. doi: 10.1002/vms3.1282 (PMC10650226; doi:10.1002/vms3.1282)
Supplement: Supplementary file 3 — Supporting Information [file VMS3-9-2559-s006.docx]

Figure S3: Monthly cases of rabies in animals in Ghana in 2012.
