# Supplementary figures and images for: Spatio‐temporal investigation of reported cases of animal rabies in Ghana from 2010 to 2017
Source: Vet Med Sci. 2023 Sep 23;9(6):2559–65. doi: 10.1002/vms3.1282 (PMC10650226; doi:10.1002/vms3.1282)

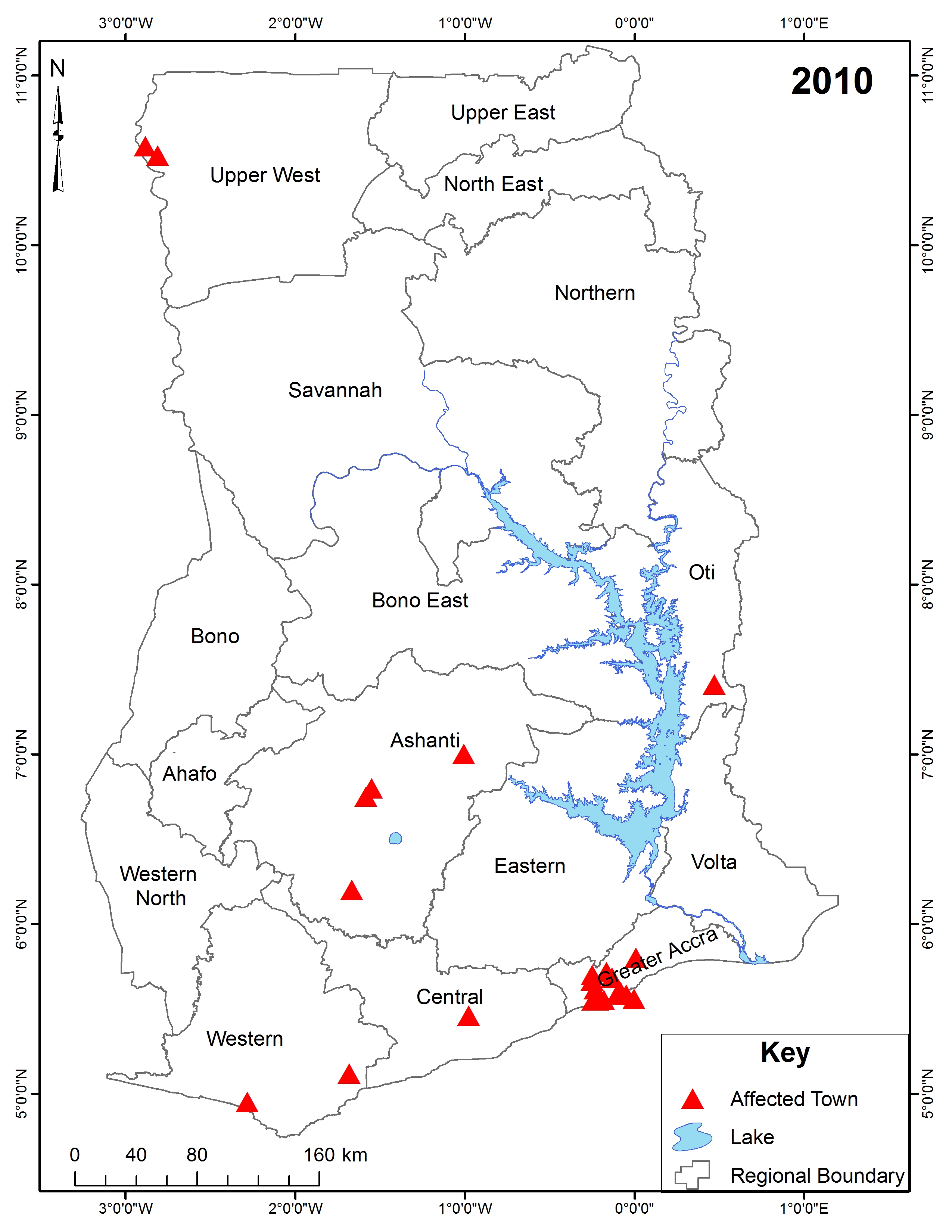

Supplement: Supplementary file 9 — Supporting Information [file VMS3-9-2559-s008.jpg]

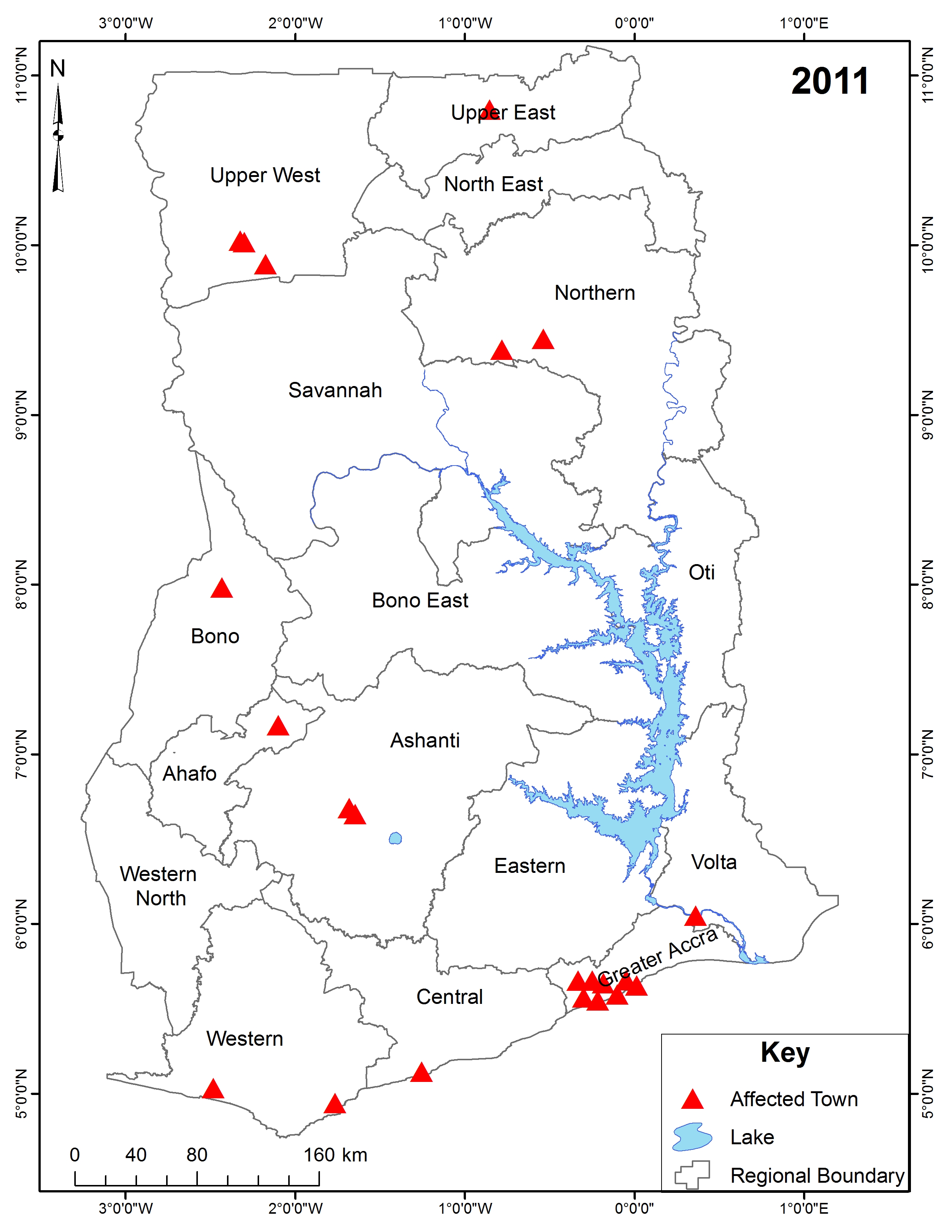

Supplement: Supplementary file 10 — Supporting Information [file VMS3-9-2559-s014.jpg]

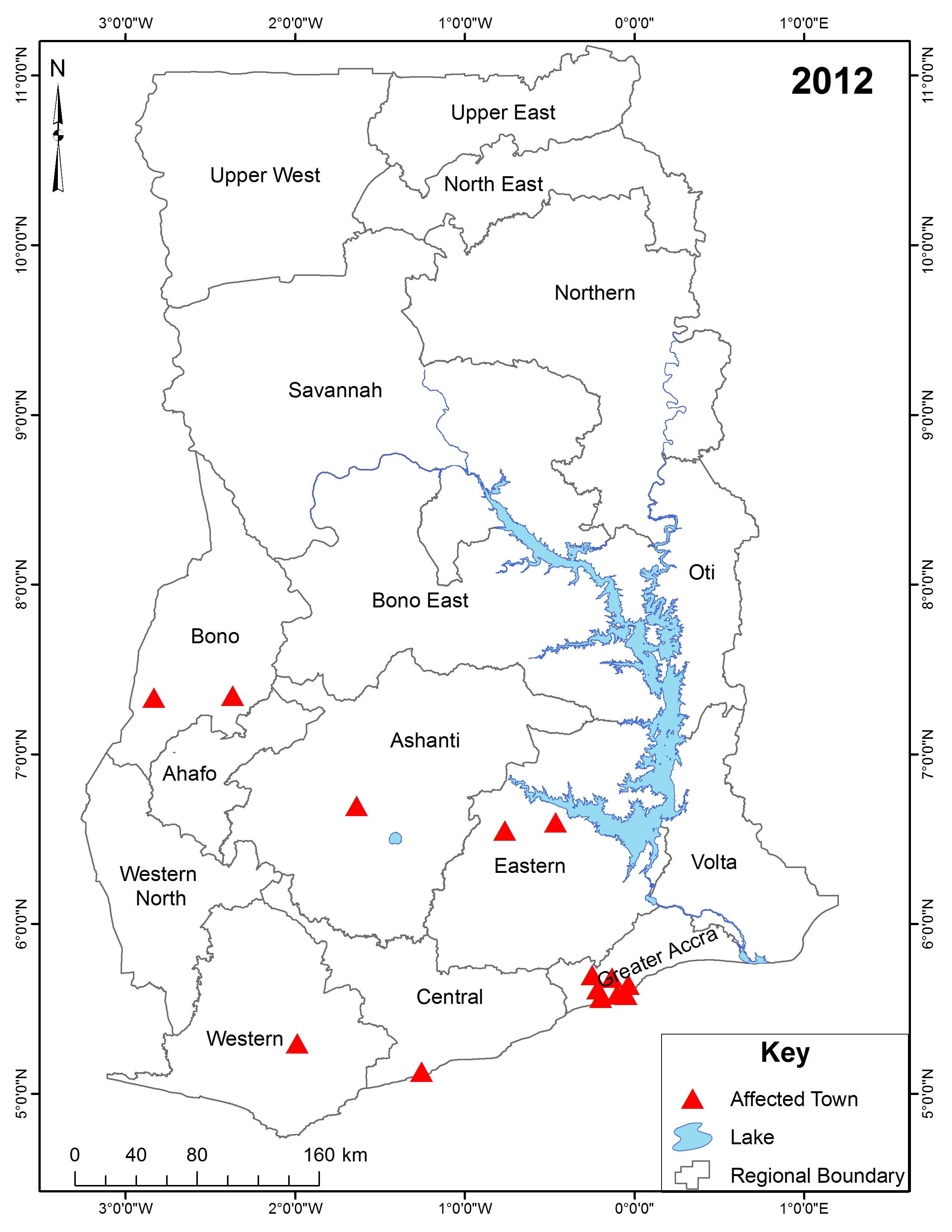

Supplement: Supplementary file 11 — Supporting Information [file VMS3-9-2559-s013.jpg]

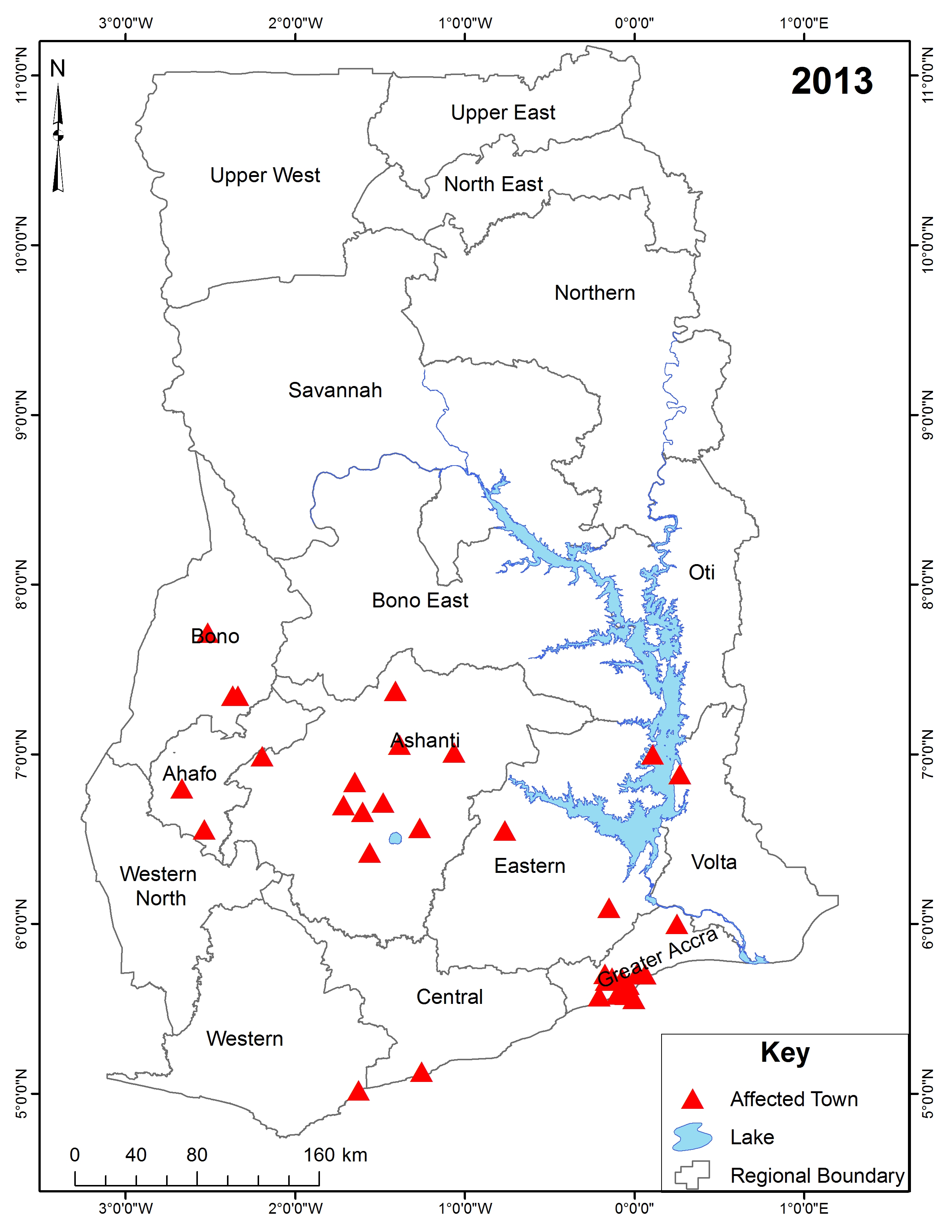

Supplement: Supplementary file 12 — Supporting Information [file VMS3-9-2559-s009.jpg]

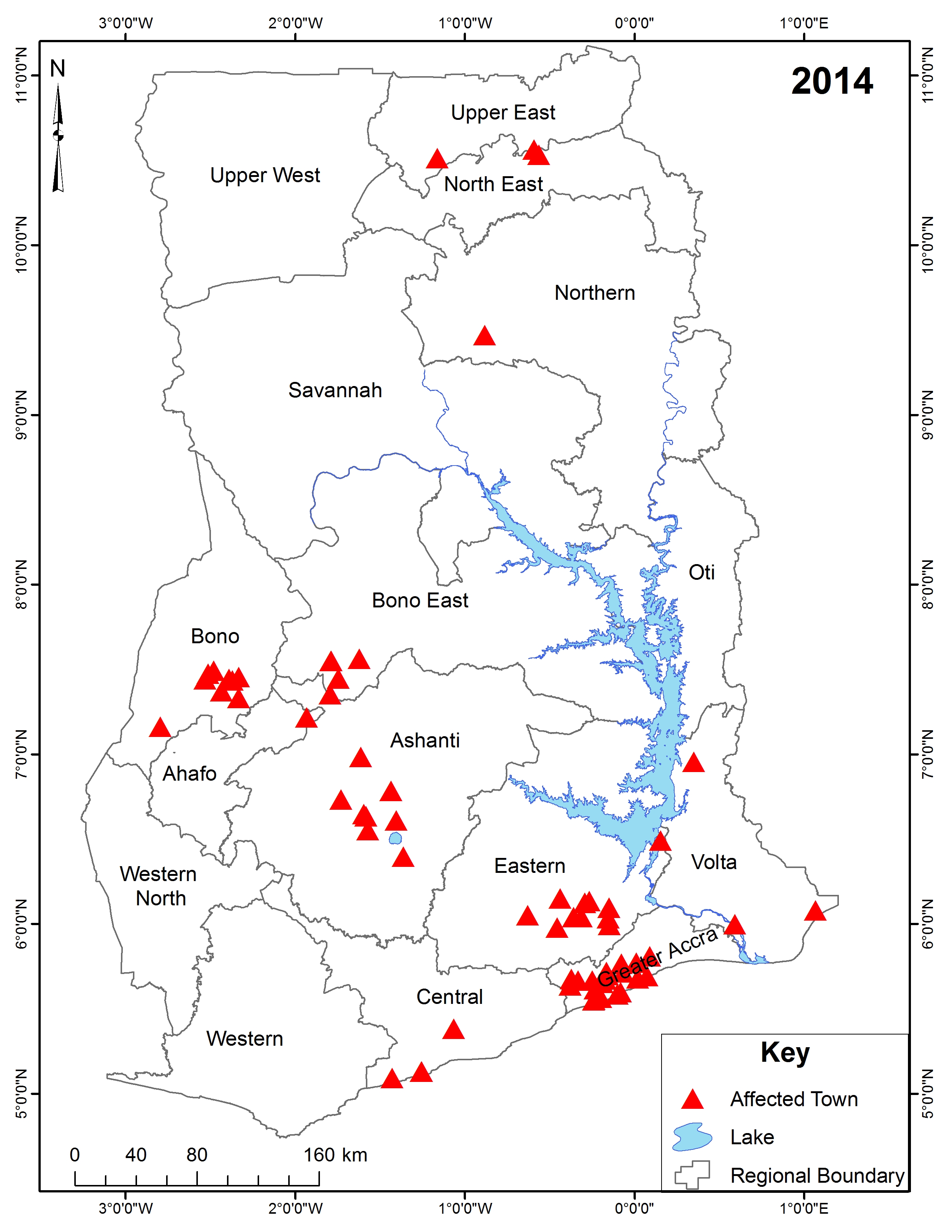

Supplement: Supplementary file 13 — Supporting Information [file VMS3-9-2559-s010.jpg]

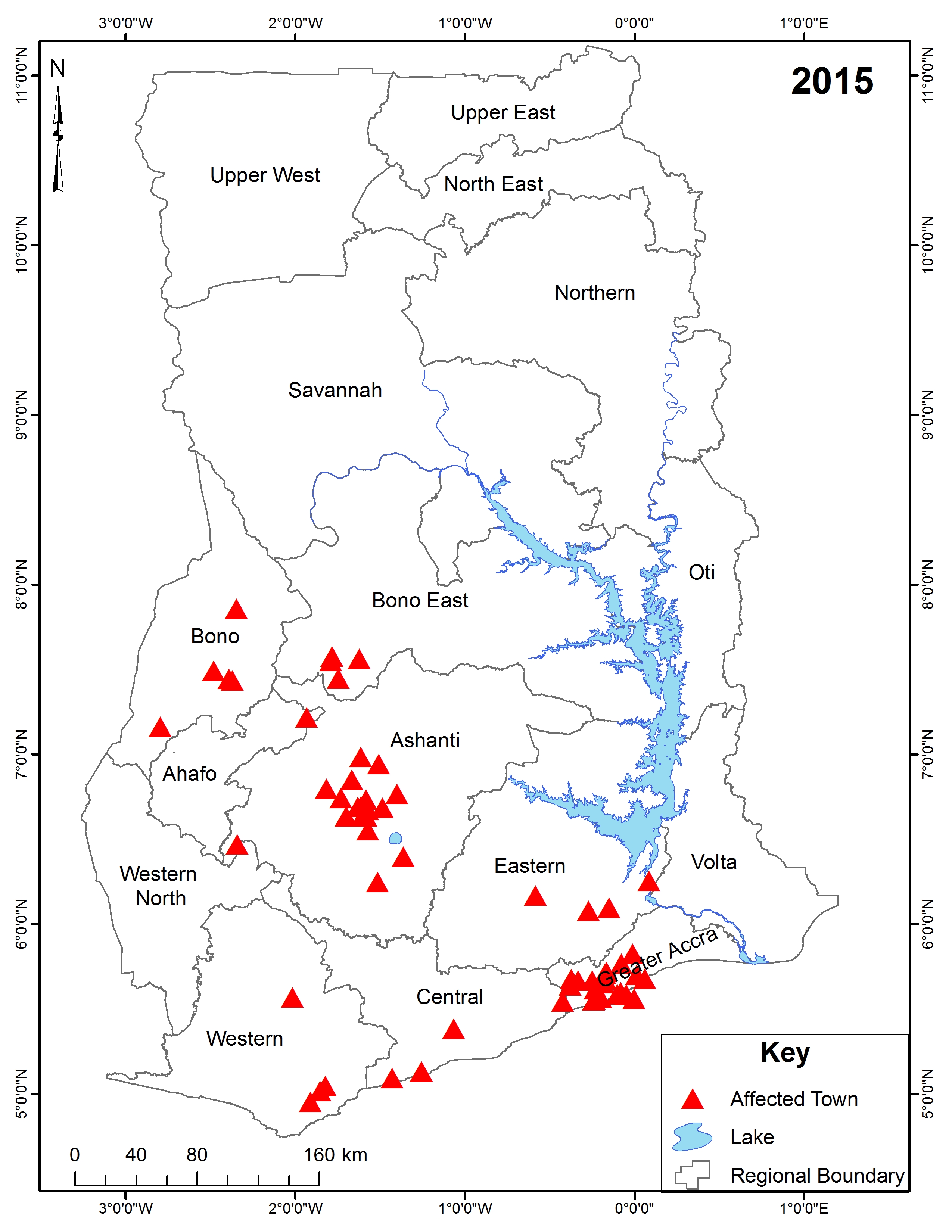

Supplement: Supplementary file 14 — Supporting Information [file VMS3-9-2559-s001.jpg]

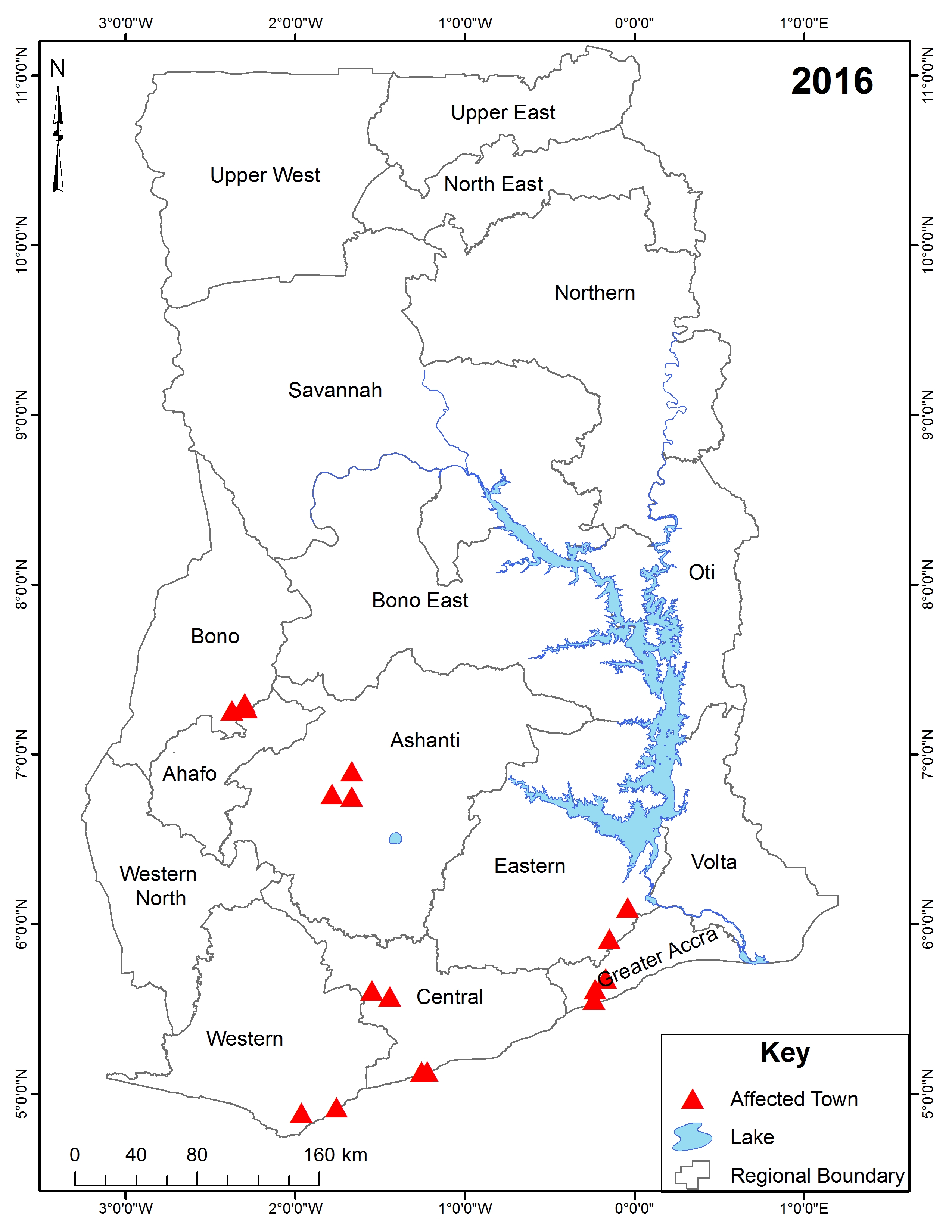

Supplement: Supplementary file 15 — Supporting Information [file VMS3-9-2559-s003.jpg]

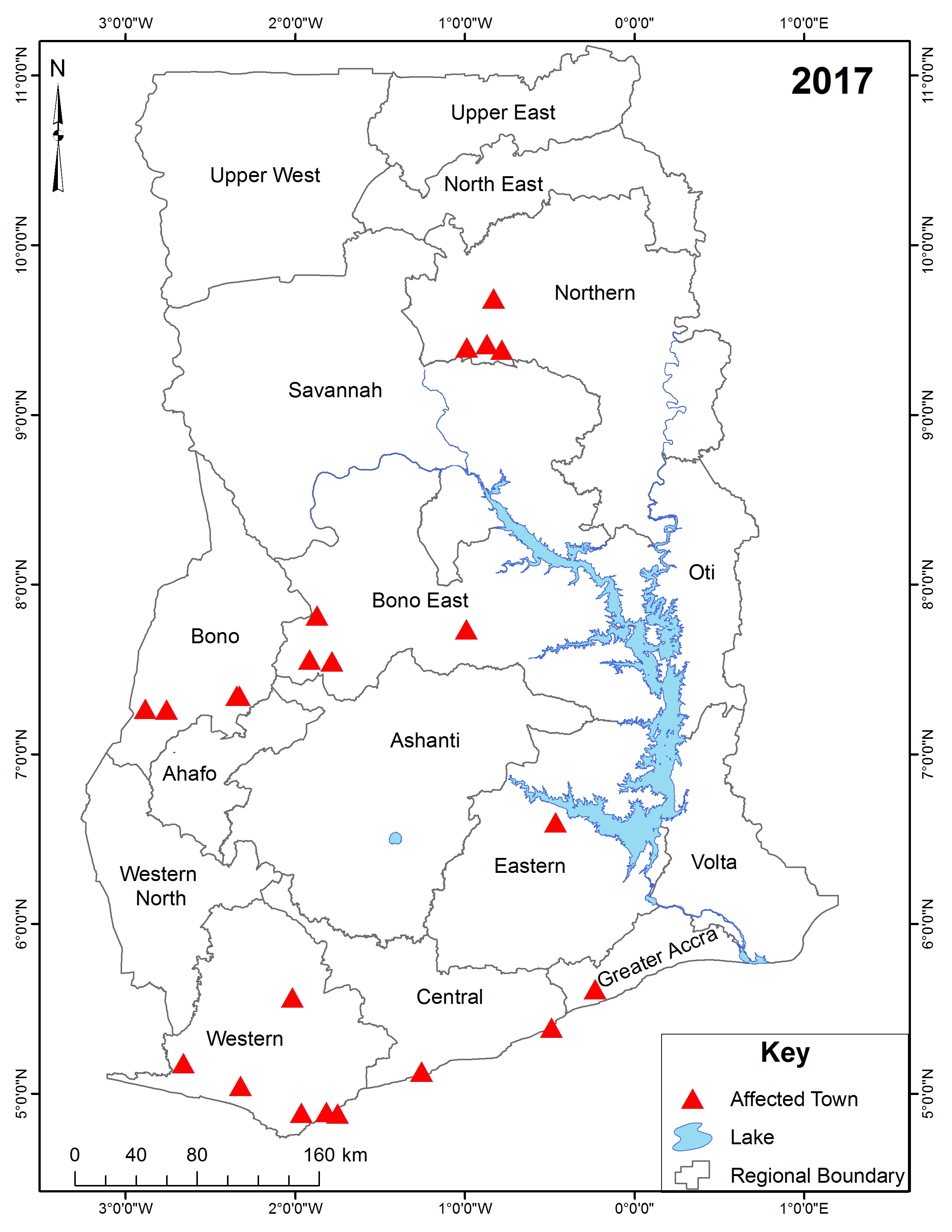

Supplement: Supplementary file 16 — Supporting Information [file VMS3-9-2559-s005.jpg]
